# Surface chemistry of rare-earth oxide surfaces at ambient conditions: reactions with water and hydrocarbons

**DOI:** 10.1038/srep43369

**Published:** 2017-03-22

**Authors:** Elçin Külah, Laurent Marot, Roland Steiner, Andriy Romanyuk, Thomas A. Jung, Aneliia Wäckerlin, Ernst Meyer

**Affiliations:** 1 Department of Physics, University of Basel, Klingelbergstrasse 82, 4056 Basel, Switzerland; 2 Glas Trösch AG, Industriestrasse 29, 4922 Bützberg, Switzerland; 3 Laboratory for Micro- and Nanotechnology, Paul Scherrer Institute, 5232 Villigen, Switzerland; 4 Laboratory for Thin Films and Photovoltaics, Empa — Swiss Federal Laboratories for Materials Science and Technology, Überlandstrasse 129, CH-8600 Dübendorf, Switzerland

## Abstract

Rare-earth (RE) oxide surfaces are of significant importance for catalysis and were recently reported to possess intrinsic hydrophobicity. The surface chemistry of these oxides in the low temperature regime, however, remains to a large extent unexplored. The reactions occurring at RE surfaces at room temperature (RT) in real air environment, in particular, in presence of polycyclic aromatic hydrocarbons (PAHs), were not addressed until now. Discovering these reactions would shed light onto intermediate steps occurring in automotive exhaust catalysts *before* reaching the final high operational temperature and full conversion of organics. Here we first address physical properties of the RE oxide, nitride and fluoride surfaces modified by exposure to ambient air and then we report a room temperature reaction between PAH and RE oxide surfaces, exemplified by tetracene (C_18_H_12_) on a Gd_2_O_3_. Our study evidences a novel effect – oxidation of higher hydrocarbons at significantly lower temperatures (~300 K) than previously reported (>500 K). The evolution of the surface chemical composition of RE compounds in ambient air is investigated and correlated with the surface wetting. Our surprising results reveal the complex behavior of RE surfaces and motivate follow-up studies of reactions between PAH and catalytic surfaces at the single molecule level.

Rare-earth oxide surfaces take an important role in catalysis and are used to catalyse a broad range of on-surface reactions, *e.g.* the. conversion of syngas (CO+H_2_) to alcohol, carbon monoxide (CO) oxidation, nitric oxide (NO) to N_2_ conversion and the water-gas shift reaction, as well as for oxidation/hydrogenation/dehydrogenation of hydrocarbons or alcohols^1^,^2^,^3^. RE oxide surfaces are expected to be catalytically active because RE metal atoms are prone to low energy fluctuations, *e.g.* to charge and spin fluctuations^4^. RE oxides, like many other oxides have been reported to be hygroscopic and, by thermal desorption spectroscopy (TDS), have been shown to form carbonates^5^. The catalytic activity of RE oxides, *i.e.* the conversion of hydrocarbons to CO_2_ and H_2_O^1^ including aromatic hydrocarbons^6^, was primary investigated at elevated temperatures where reacted species desorb after reaction and the surface is thereby continuously reactivated for further conversion of fresh reactants from the gas flow. There are only a few reports addressing a dissociation or reaction of small molecules such as CO_2_, CH_4_, and formic or acetic acids on RE oxide surfaces at room temperature (RT) and below^7^,^8^,^9^,^10^. To the best of our knowledge no other small molecules or higher hydrocarbons, were investigated in their reactivity with RE oxides at RT. There are inconsistent reports in the literature with regard to the surface chemistry and on-surface chemistry of RE surfaces in that i) Azimi *et al*.^11^,^12^,^13^ attributed the intrinsic hydrophobicity of RE oxides solely to the unique electronic structure of rare-earths inhibiting H-bonding between water molecules and ii) Zenkin *et al*.^14^ reported that the stronger intrinsic hydrophobicity of RE nitrides is based on the reduced number of lone pairs of electrons in the N anion resulting in an even lower ability to form H-bonds with water. Recently, the effect of ambient air-exposure modifying the hydrophobic properties of RE oxides had been reported: the observed simultaneous increase of carbon at the surface has been related to an increase of the hydrophobicity^15^, but further mechanistic insight has not been provided into the surface and on-surface chemistry involved in this process.

Here we address (i) the water induced evolution of RE containing surfaces with different polarity which depends on the Pauling electronegativity difference (Δχ~2.3 for oxides, Δχ~1.9 for nitrides and Δχ~2.9 for fluorides) of the composing ions. Such highly polar surfaces are expected to strongly interact with water molecules^16^,^17^,^18^,^19^. In this light, the recently reported intrinsic hydrophobicity^11^ is very surprising (Fig. 1a). Also, (ii) we analyse the surface chemistry of RE oxides and correlate it with the hydrophobicity stemming from the exposure to ambient air. Finally, (iii) we report the first example of rare-earth oxides promoting the oxidation of the polycyclic aromatic hydrocarbon tetracene (Tc, C_18_H_12_) at low surface coverages.

## Results

**Figure 1:**
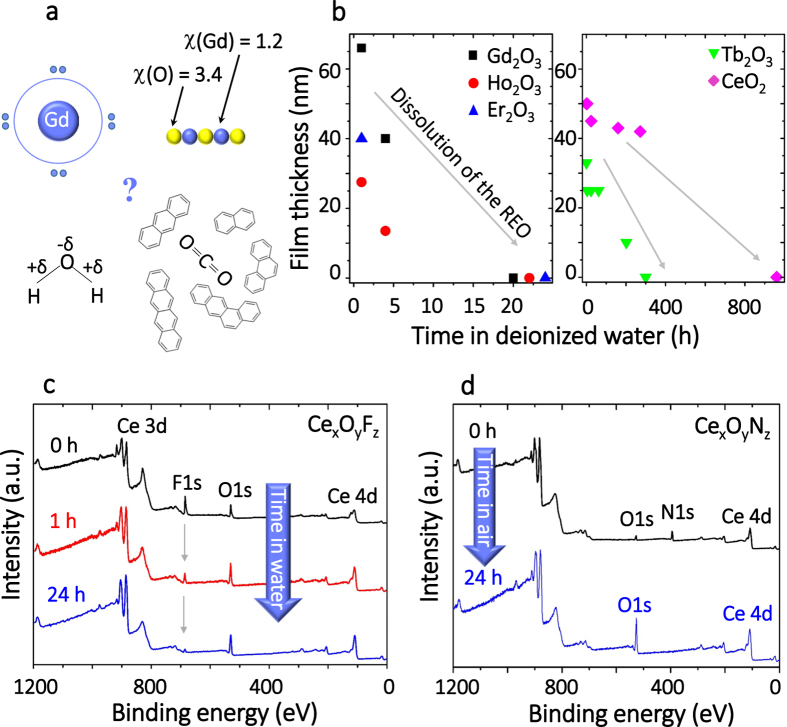
Reaction of RE oxides, oxy-fluorides, and oxy-nitrides with water. (**a**) Schematic illustration of the factors impacting the surface chemistry of RE oxide surfaces upon air exposure, regarding the origin of the earlier reported hydrophobicity^11^,^12^. (**b**) Exposure of Gd, Ho, Tb, Er, Ce oxide films to 1 liter of deionized water leads to dissolution of the films as evidenced by the profilometer thickness and XPS measurements (Supplementary Fig. S1). This data confirms the ionic nature of the RE oxides, which are prone to dissolve in water. (**c**) Exposure of Ce oxy-fluoride films to 1 liter of deionized water leads first to the substitution of F by O and then to dissolution, as evidenced by XPS. Furthermore, Ce oxy-nitride undergoes substitution of N by O even without dipping into water, but only by being exposed to air for less than 24 h.

In order to investigate the modification of RE derived oxide, nitride and fluoride surfaces by their exposure to water and volatile organic compounds, thin film samples (30–80 nm) on SiO_2_ surfaces were prepared in vacuum by magnetron sputtering (2 cm × 4 cm in size, see methods section). The thin film samples were then dipped into 1 liter of deionized water at RT for a defined time. Afterwards the thickness of the deposited film was measured by profilometry and the chemical state of the films was studied by X-Ray Photoelectron Spectroscopy (XPS). In cycles of exposure and experimental investigation, the surface adlayer formation and the dissolution kinetics of the Gd, Ho, Tb, Er and Ce oxides were monitored (Fig. 1b, Supplementary Figs S1, S2). Gd_2_O_3_, Ho_2_O_3_, Er_2_O_3_ dissolved within 24 h, and Tb_2_O_3_ and CeO_2_ dissolved within 1 month or less. The dissolution process of RE oxides proceeds in the following steps: i) dissociation/reaction with H_2_O and formation of hydroxide layers and ii) dissolution of the material^16^. Note that the solubility of RE ionic compounds in water ranges between 10^−6^ and 10^−5^ mol/L^16^,^17^. This solubility is sufficient to dissolve ~120–1200 ML of RE oxide covering our sample (2 × 4 cm^2^) in 1 L of water which provides a limit to practical applications of RE oxides as functional coatings at atmospheric conditions.

Following up on the surprising observation of the surface chemical modification of RE oxides, we have extended our investigations also to RE fluorides and nitrides. According to Zenkin *et al*.^14^, nitrides of REs exhibit an even larger hydrophobicity than RE oxides. Due to the high reactivity of the nitride films towards water and oxygen (see below), small amounts of oxygen were detected by *in-situ* XPS even directly after preparation (see Supplementary Table S1). Therefore the fluoride and nitride films are hereafter referred to as oxy-fluoride and oxy-nitride.

Dipping of cerium oxy-fluoride film Ce_x_O_y_F_z_ into 1 liter of deionized water for defined periods of time: *i.e.* 1 and 24 h, changed the surface composition of the film drastically: the concentration of F decreased and the concentration of O increased, as seen by XPS (Fig. 1c, Supplementary Table S1). The thickness of the film did not change noticeably at the same time. The release of F^−^ ions from CeF_3_ upon water contact along with the subsequent Ce hydroxide formation has been reported earlier^20^. The 10 nm film of Gd_x_O_y_F_z_ in comparison to 10 nm film of Ce_x_O_y_F_z_ dissolved much faster, *i.e.* within 17 h (*cf.* Supplementary Fig. S2, Supplementary Table S1). The oxy-nitrides (Ce_x_O_y_N_z_) appear to be very unstable: Substitution of N by O atoms in RE nitride occurs readily in air^21^ as confirmed by the absence of the N1s signal in XPS after 1 day of exposure to air (Fig. 1d and Supplementary Table S1). We note that all these materials *i.e.* the oxides and the nitrides, which are readily converted to oxides, are water soluble and as such are not hydrophobic.

**Figure 2:**
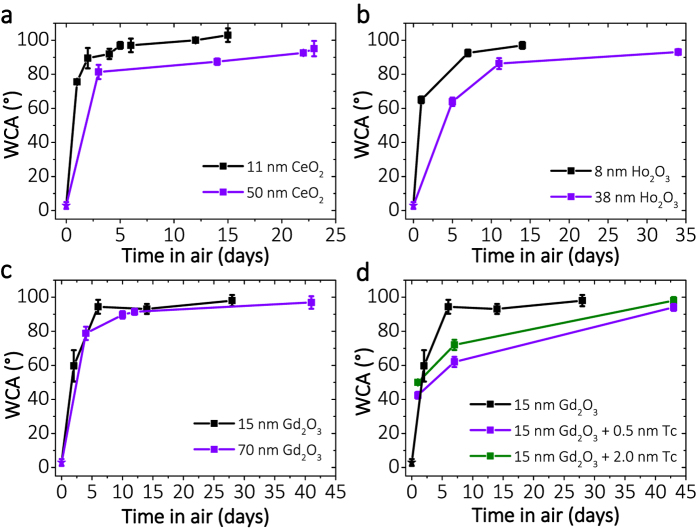
Time-dependent evolution of hydrophobicity in air of pure RE oxide and adsorbate-modified RE oxide surfaces. Water contact angle (WCA) measurements for two different thicknesses of (**a**) CeO_2_, (**b**) Ho_2_O_3_, (**c**) Gd_2_O_3_ and (**d**) thin films of tetracene at Gd_2_O_3_ surfaces (compared to native Gd_2_O_3_). Low WCA values are measured within the first 24 h after film preparation. The contact angle increases with increasing exposure time. This provides evidence for a slow adsorption mechanism modifying the surface coverage and contact angle. The stars denote the ‘initial’ WCAs, measured directly (within 1 min) after annealing aged films to 600 °C (see text). Each WCA curve exhibits two different slopes (‘steep’ within the first 7 days and ‘less steep’ afterwards) evidencing process which involves either two different kinetic regimes of the same or two different processes and/or more than one involved compound (see XPS discussion).

In addition to the long term solubilisation experiments of the RE oxide, fluoride, and nitride surfaces in water, we investigated the evolution of hydrophobicity/hydrophilicity of Gd_2_O_3_, Ho_2_O_3_, Er_2_O_3_, Tb_2_O_3_ and CeO_2_ surfaces upon exposure to ambient air. For this purpose the water contact angle (WCA) in dependence of the duration of ambient air exposure was measured. The RE oxides exhibit low WCAs (40–60°) after 1 day of being in air, and then exhibit increasing WCAs to 90–100° over time (Fig. 2). Interestingly, we also observe that the exposure time to reach a hydrophobic state depends on the film thickness. The surfaces of thinner films transform faster to hydrophobic states (high WCA) than thicker films (Ho_2_O_3_ - Fig. 2a, CeO_2_ - Fig. 2b, Gd_2_O_3_ - Fig. 2c: violet - thick *vs*. black - thin). RE oxide surfaces of films with different thicknesses reach similar final WCAs in the range of 95–100° over time. Note that the hydrophobicity of RE oxides in our studies does not depend on the storage time in vacuum, in contrast to the earlier report of Khan *et al*.^11^, see discussion in Chapter 3B of Supplementary Information for the case of cerium oxide. The dependence of the WCA on the air exposure time suggests the modification of the surface by non-polar, low surface-free-energy volatile organic compounds (VOC) adsorbed from ambient air^15^, as reported for transition metal oxides^22^, as well as for graphitic surfaces^23^,^24^. There are various types of volatile hydrocarbon molecules present in air^25^,^26^,^27^ at different concentrations depending on the urbanization level. Thereby they are also available for adsorption at the RE oxide film surface. The existence of an adsorbed layer is evidenced by heating of the aged-in-air RE oxide surface for 4 minutes at ~600 °C (ramping-up, tempering and cooling down to RT in a total time scale of 20 min) which rendered the film hydrophilic (WCA<5°) when measured within 1 min after heating (data points marked by stars in Fig. 2). This observation is consistent with the conversion of hydrocarbons to CO_2_ and H_2_O at elevated temperatures^5^ and their thermal desorption from the surface.

In order to verify the impact of low coverage airborne hydrocarbons on the hydrophobicity of the film, we have selected a model example of a polycyclic aromatic hydrocarbon, namely tetracene. The molecule was thermally evaporated *in-situ* onto a freshly prepared Gd_2_O_3_ film kept in vacuum (see Methods). One deposition was estimated to be in the order of 0.5 nm thickness (Fig. 2d, red) the other, in the order of 2 nm (Fig. 2d, black). The resulting coverage is estimated to be in the order of 1–2 monolayers on the basis of XPS, but is not trivial to determine because i) the adsorption probability is not equal to 1, ii) the surface of the Gd_2_O_3_ is not atomically flat, iii) there are different possible orientations (horizontal or perpendicular to the surface) of the molecules within the layer (lying flat or on-standing configuration). The chemical state of Tc and of the RE oxide film as well as the amount of adsorbate was probed immediately after deposition of Tc by XPS (see *vide infra*) without breaking the vacuum. The WCA and non-contact atomic force microscopy (nc-AFM) measurements were performed *ex situ*, directly after their removal from the growth chamber, the next day. The Tc covered surface was not initially hydrophobic and was additionally observed to be retarded in its evolution towards hydrophobicity upon air exposure compared to native Gd_2_O_3_ substrate (Fig. 2d).

**Figure 3:**
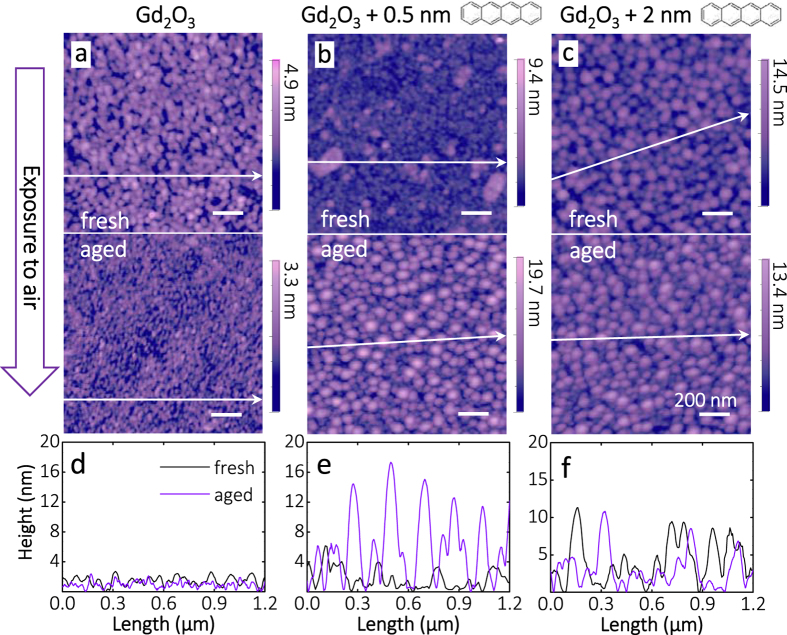
Modification of the RE oxide surfaces by adsorption of hydrocarbons: Morphology. Three different conditions of the Gd oxide surface were investigated by non-contact atomic force microscopy in its (**a**–**c**, top) fresh state and in its (**a**–**c**, bottom) >1 month aged and hydrophobic state. As evidenced by the AFM micrographs (1.3 × 1.3 μm^2^) and their cross-sections (**d**–**f**: black – fresh, violet – aged), deposition of tetracene onto Gd_2_O_3_ significantly modifies the morphology of the film (see Table 1 for rms roughness and lateral correlation length). The surface of Gd_2_O_3_ undergoes different morphological changes depending on the starting conditions, *i.e.* native *vs*. Tc covered films. Moreover, exposure to air changes not only the morphology of each system but also the chemical composition (Fig. 4) and the resulting WCA (Fig. 2d).

The morphology of the films after 1 day in air, in its ‘not-yet hydrophobic’ state and after prolonged exposure to air, in its hydrophobic state, has been investigated by AFM. The morphology of the native Gd_2_O_3_ film (Fig. 3a) changes upon deposition of Tc (Fig. 3b,c), as reflected by increase in vertical roughness, root mean square (RMS) values, with increase of Tc coverage (Table 1). The formed grains/islands reach up to 10 nm in height (Fig. 3e,f). Tetracene is known to self-assemble into a parallel stacked structure upon increasing the coverage^28^, thereby at a certain coverage a close-packed layer of molecules oriented in ‘out-of-the-surface-plane’ orientation can be expected in analogy to the crystal structure of tetracene^29^. Lateral correlation length is higher of Tc covered films reflecting bigger grain size, as can be also observed from AFM images and corresponding cross-sections (Fig. 3d–f). Lateral correlation length was defined from fit of Height-Height Correlation Function (see chapter 6 of Supplementary Information)^30^. Aging in air reduces the correlation length slightly. Prolonged exposure of Tc/Gd_2_O_3_ surfaces to air results in higher surface roughness compared to the aged native Gd_2_O_3_ film. All RMS values and lateral correlation lengths are summarized in the Table 1. It is important to note that the surface morphology of the exposed to air surfaces is less stable with regard to scanning induced modifications in the AFM than freshly prepared Gd_2_O_3_ or Tc/Gd_2_O_3_ surfaces. Thus, after aging, AFM imaging depends on mild scanning conditions (see details in Methods). We take this as evidence for the presence of adsorbates at the surface. Significant change in WCA upon aging in air evidences the modification of the top surface layer.

**Figure 4:**
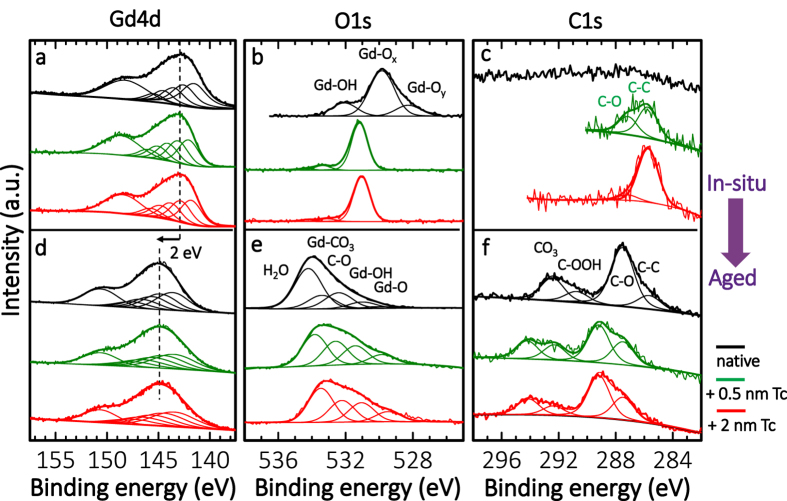
Reactivity of Gd oxide towards Tc and progressive modification of the surface chemical composition in air: XPS analysis. Normalized Gd4d, O1s, C1s XP spectra for *in-situ* (**a**–**c**) and air-exposed (**d**–**f**) surfaces are compared for: 1) native Gd_x_O_y_ (black), 2) Tc-modified Gd_x_O_y_ surface at two coverages −0.5 nm (green) and 2 nm (red). *In-situ* deposition of Tc onto Gd_x_O_y_ surface at RT induces a remarkable increase in BE of O1s by 1.3 eV, as well as decrease in total oxygen concentration with increase of Tc coverage evidencing a reduction of the RE oxide surface (see Table 2). Moreover, the C1s spectra reflect two carbon species: i) the C-C bonds of the molecule and ii) the carbon of the molecule which participates in the C-O bonds formed at the molecule/surface interface. Induction of C-O bonds along with reduction of Gd_2_O_3_ surface evidences a reactivity of rare-earth oxide towards hydrocarbons at RT. Exposure to air and aging leads to a significant change in the surface chemical composition of the film. This process occurs for all three RE oxide systems (see discussion in the text).

In order to address the surface chemistry occurring at the RE oxide interface induced by Tc deposition, as well as ageing induced modifications during air exposure, we employ XPS (Fig. 4). Gd_2_O_3_ surfaces have been prepared by sputtering *in-situ*, and investigated before and after i) controlled exposure to air at ambient pressure and ii) *in-situ* exposure to aromatic tetracene, Tc (Fig. 4, top part: *in-situ*) and in subsequent aging steps in air (Fig. 4, bottom part: aged). The Gd 4d spectra exhibit core-level splitting caused by spin-orbit coupling^31^ with the strongest spectral features (^9^D and ^7^D final ionic states) stemming from the interaction of 4d photohole with the 4f^7^ valence band electrons^32^. Consequently, 6 components were fitted with the defined parameters of relative FWHM, relative intensities, and relative energy positions^33^,^34^. In order to analyse the chemical state of the film surface all the XP spectra taken from the films *before* exposure to air (Fig. 4a–c) were charge-corrected with reference to the binding energy of the Gd 4d ^9^D peak at 142 eV^35^, and fitted with the minimum number of components (Fig. 4a). The native, *in-situ* prepared film before exposure to air exhibits a dominant O1s peak (Fig. 4b, black) at 529.8 eV corresponding to the formal Gd(3+) oxidation state (stoichiometric Gd-O_x_, Gd_2_O_3_), as well as a shoulder located at lower BE (528.3 eV) corresponding to the more oxidized Gd-O_y_ form, which we attribute to excess O and lattice defects, plausible in the sputtered amorphous film. A slight excess of O is also revealed in the atomic concentration analysis, where a ratio of total concentrations O to Gd is 2:1 (Table 2). Notably, also the special chemistry of rare earth elements allows for many different states of the f-shell and contributes to a wide range of stoichiometries^36^, which can vary depending on the sputtering process.

A higher concentration of O in the O:Gd ratio moves the O1s BE to the lower side, and a lower concentration of O moves the O1s BE to the higher side (vice versa for the Gd BE)^37^. In our analysis we chose the Gd4d ^9^D peak as the reference. Therefore only the positions of the O1s, C1s peaks shift in our spectra. The small peak at its highest BE of 532.1 eV, corresponds to a Gd-OH bond^38^ which is formed due to a small amount of water present in the experimental chamber. The native film is free of any C contamination, as it is evident in the C1s spectrum (Fig. 4c, black).

*In-situ* evaporation of a few MLs of Tc (C_18_H_12_) onto a Gd_2_O_3_ surface at RT sheds light onto the reactivity of RE oxides towards aromatic VOC. First, 0.5 nm of Tc was evaporated during 2 min resulting in a total atomic concentration of carbon of 19.9%. The C1s core level spectrum was de-convoluted in two singlets: C-C bonds at 285.7 eV^29^ (12.5%) – stemming from Tc and C-O bonds at higher BE (+1.4 eV)^39^,^40^,^41^. This analysis evidences the formation of C-O bonds (7.3%) at the molecule/surface interface. A 4-times increase in Tc coverage, according to the quartz crystal microbalance, resulted in less than 2-times increase of the total surface carbon concentration to 28.1%, with 25.6% C-C bonds (see Supplementary Table S2), evidencing adsorption/desorption processes occurring during Tc evaporation. The amount of C-O bonds (2.5%) detected by XPS is smaller at higher Tc coverage. This is consistent with the limited penetration depth of the photoelectrons through the layer, as the C-O bonds originate from the interaction with the substrate. Strikingly, Tc evaporation induces a decrease in total concentration of O with an increase of Tc coverage: i) for a native Gd_2_O_3_ is O : Gd = 2 : 1, ii) for 0.5 nm Tc/Gd_2_O_3_ is O : Gd = 1.7 : 1, iii) for 2 nm Tc/Gd_2_O_3_ is O : Gd = 1.4 : 1 (Table 2). Significant shift in O1s core level spectrum, towards higher BE (+1.3 eV with respect to O1s peak of the native substrate Gd-O_x_, Gd_2_O_3_) occurs simultaneously. Loss of oxygen from the surface and increase in BE of O1s evidences a reduction of the Gd_2_O_3_ surface^42^ upon deposition of Tc and hints on the reactivity of surface towards Tc already at RT. We underline that so far only reactions of light hydrocarbons with RE oxide surfaces were reported in the literature^9^,^10^. The mechanism of C–O bond formation, *i.e.* cleavage of C–H bond in Tc and its partial oxidation is tentatively assigned to reactivity of O^−^ species stemming from the surface and lattice defects, as well as reactivity of lattice oxygen O^2−^, leading to formation of OH^−^ and/or to reduction of the oxide surface supplemented by the consumption of oxygen from the surface (if partially oxidized molecule desorb), as was reported for partial oxidation of methane CH_4_, ethylene C_2_H_4_ and other hydrocarbons at REO at elevated temperatures^3^,^9^,^43^.

Moreover, the reduction of native Gd_2_O_3_ surface after Tc deposition can be correlated also with the increased surface roughness observed for the Gd_2_O_3_+ Tc system vs. native Gd_2_O_3_ in the AFM images (Fig. 3b,c vs. Fig. 3a), *i.e.* etching of the surface by consumption of oxygen. Note, the C1s BE of aromatic hydrocarbons can further be influenced by direct contact to the substrate and by the orientation of the molecules^29^. On oxide surfaces Tc is expected to be up-right standing at RT^29^.

The impact of air exposure on the surface chemistry of a clean Gd_2_O_3_ film surface, as well as of Tc-covered Gd_2_O_3_ is analysed in the following: By exposure to air the RE oxide surface gets in contact with H_2_O, CO_2_, and airborne hydrocarbons. XPS measurements with progressive increase of air exposure reveal a drastic change in the surface composition (Fig. 4e,f). RE oxides readily react with water^38^ forming bulk hydroxide, and CO_2_ forming top layers of carbonate^5^,^44^,^45^,^46^. This was also observed by dosing of CO_2_ onto CeO_2_ surfaces which caused the formation of Ce_x_(CO_3_)_y_ at temperatures as low as 180 K^48^. As Gd_x_(CO_3_)_y_ is formed on the surface after exposure of Gd_2_O_3_ to air, the BE for Gd4d was moved towards higher BEs (ca.+2 eV with respect to Gd 4d in the non-exposed oxide, *i.e.* to 144 eV) in the charge correction. This increase in BE is expected as metal carbonates generally have a higher cation BE than metal oxides^48^.

In all cases of Gd_2_O_3_ surfaces aged in air, native (Fig. 4f, black) or Tc-covered (Fig. 4f, red, green) C1s core level spectra were deconvoluted into the same four singlets evidencing a similar surface chemical composition. The first peak at the lowest BE is attributed to C-C bonds, each subsequent peak placed at +1.8 eV, +5.0 eV, and +6.7 eV corresponds to C-O, C-OOH and C-O_3_ related species respectively^47^,^49^. Strikingly, the two major components in C1s spectra of aged in air Gd_2_O_3_ (at 286.5 eV and 291.5 eV) and Tc-covered Gd_2_O_3_ (at 289.2 and 294.2 eV) have the same peak-to-peak distance of ΔE ~5 eV and the same ratio: 1 : 2.6 for native Gd_2_O_3_, 1 : 2.6 for 0.5 nm Tc/Gd_2_O_3_, and 1 : 2.7 for 2 nm Tc/Gd_2_O_3_. The ratio is preserved for all the aged samples and corresponds to C-O present at the interface and CO_3_^2−^ bonds present in the carbonate layer. At RT no physisorbed CO_2_ species at RE oxide surfaces are expected, as CO_2_ dissociates and/or reacts with RE oxides by forming carbonate even at lower temperatures^47^. O1s core level spectra were deconvoluted into four singlets at 530 eV (+1 eV, +2.4, +3.6 eV, +4.3 eV), corresponding to the Gd-O, Gd-OH, C-O, COOH, and CO_3_^2−^ derived phases^50^ (Fig. 4e). Appearance of C-C, C-O, and C-OOH bonds on the native Gd_2_O_3_ surface after exposure to air (Fig. 4c *vs*. Fig. 4f - black) evidences an adsorption of airborne hydrocarbons. On the one hand, the final total concentration of carbon on both the native and Tc pre-covered Gd_2_O_3_ surfaces is similar (~25%). The Tc pre-covered Gd_2_O_3_ surface exhibited a significant reduction in the concentration of C-C bonds after exposure to air (0.5 nm Tc film: from 12.5% to 6.1%, the 2 nm Tc film: from 25.6% to 6%, see Supplementary Table S2) hinting towards the conversion/decomposition of Tc, and the self-limited establishment of an adsorbate layer or – more probably – a steady state situation of adsorption, reaction and desorption processes in air (Fig. 4c *vs*. Fig. 4f – green, red). Interestingly, the final surface concentration of C-C bonds in the aged Tc films is nearly the same indicating that the saturated composition of the surface is independent from the starting amount of Tc on the surface. However, the exact role of water and excess oxygen in the surface chemical reaction between hydrocarbons (tetracene) and the RE oxide surface is not totally clear. The process is complex, nevertheless, one can distinguish at least 4 steps which occur simultaneously: Gd_2_O_3_ reacts with H_2_O forming Gd-OH^46^ and with CO_2_ forming Gd-CO_3_^39^,^46^. With hydrocarbons it reacts by partially or fully oxidizing them and forming C-O bonds until a saturated carbonate layer is formed. After all RE atoms have undergone a surface reaction and are saturated as the carbonate layer the reaction rate with organic deposits (Tc in our case) is lower. This rate change is consistent with the time dependent evolution dynamics of the WCA (Fig. 2). Surface chemical reactions with airborne hydrocarbons, hydroxylation and carbonation, take place on all rare-earth films and are also exemplified here by CeO_2_ exposed to air. Exposure of the surface for prolonged times to air induces a significant modification in the 3d core level of Ce in the surface proximal layer (*cf*. XPS in Supplementary Fig. S3). The oxidation state of the RE ion inside the film is converted from (almost) 4+ to a mixture of 3+ and 4+ providing further evidence for the surface chemical evolution described here.

In the context of hydrophobicity, any carbonate as an ionic compound, strongly interacts with water and is hydrophilic^51^. Therefore the observed high water contact angles (Fig. 2) after short term water exposures can only stem from non-polar airborne organic adsorbates. In the long term, water begins to dissolve the RE carbonate/hydroxide surfaces^16^,^17^,^52^ and leaves a hydrophilic RE oxide top layer. It is important to note that the surface takes one week in air in order to become hydrophobic. This points at two necessary steps in the surface chemical transition: i) the saturation/passivation of Gd^3+^ bonds by CO_3_^2−^, the formation of which is induced by adsorbed water, CO_2_, and any present hydrocarbons; ii) the adsorption of hydrophobic hydrocarbons onto a carbonate passivated RE oxide surface. Aerosols in the atmosphere of the urban eco-system include VOCs from natural and anthropogenic sources, which include: non-methane organic gases, polycyclic aromatic hydrocarbons (PAH), and carbonyls of size less than 20 C-atoms^27^,^28^. Even though VOCs in air are of low concentrations, air exposure for about a week ensures a full monolayer at the surface. The presence of O = C-O bonds, as detected in C1s and O1s signals, can also be assigned to carbonyl functional groups present in the adsorbed organic species. These anchor by deprotonation at the surface of RE oxides^8^ leaving an up ward standing hydrophobic tail, as shown earlier for fatty acids on CaCO_3_ surfaces^51^,^53^. Additionally, C-O bonds may also stem from the C-O-C ether backbone of the adsorbate.

## Conclusions

We have shown that exposure of rare-earth oxide, fluoride, and nitride surfaces to ambient air modifies the physical properties of the surface, namely reduces wetting by water and increases roughness. The air exposure induces changes of the surface chemical composition by the adsorption of volatile organic compounds. Detailed investigations of the Gd_2_O_3_ surface demonstrate the reactivity of the Gd oxide surface towards volatile hydrocarbons from the ambient air and their partial oxidation already at RT, as exemplified by tetracene adsorbates. Extended ambient air exposure (aging) of RE films leads to a conversion of the topmost RE oxide layers to carbonate and hydroxide, as well as to a self-limited accumulation of volatile organic compounds. The long time required to build-up hydrophobic layers in ambient air suggests that volatile organic compounds adsorbed onto the surface react with RE oxides and form a carbonate until the surface is covered. The fact that also thick layers of tetracene (Tc) got converted strongly suggests a continuous evolution of surface with adsorption, reaction, desorption and release of the involved compounds.

## Methods

### Sample preparation

The deposition of the RE oxide was performed in a HV system with a base pressure of ~10^−7^ mbar by magnetron sputtering. Stoichiometric (CeO_2_, Gd_2_O_3_, Ho_2_O_3_, ErO_2_, Tb_2_O_3_), under stoichiometric (Gd_x_O_y_, Ho_x_O_y_), and metallic (Ce) targets of diameter 2.5 cm were used. The films were grown on a 4 mm thick float glass, covered with 45 nm of SiO_2_. Typical sputtering process for oxides was conducted with a 35 sccm Ar flow and 50–80 W power supply (DC −250 kHz frequency, 2456 ns pulse time/RF – bias voltage ~300 V). Investigated film thicknesses were in the range of 8–90 nm. RE fluorides and RE nitrides were prepared by adding CF_4_ or N_2_ gas during the sputtering process. A gas mixture of 70 sccm Ar and 1 sccm CF_4_ was used for the fluorides, operating at 70 W. Nitrides were deposited with 35 sccm Ar and 10 sccm N_2_ at 40 W. Total gas pressure during sputtering was in a range of 10^−3^–10^−2^ mbar.

The deposition rate was measured by quartz-crystal microbalance (QCMB), and was in order of 0.05–1.2 Å/min. The thickness of the film was cross calibrated on the basis of additional ellipsometry and profilometry measurements.

Tetracene was deposited *in-situ* by means of thermal evaporation at ~230 °C (p_evap_ ~9 × 10^−8^ mbar) onto freshly prepared Gd_2_O_3_ films. The tetracene molecules were well outgassed at a constant evaporation rate during ca. 2 h prior the experiment. The evaporation rate and deposition time was controlled by QCMB. The total deposition time was below 10 min.

### Film characterization

The RE oxides were transferred *in-situ* to the XPS chamber directly after deposition. The pressure in the XPS chamber was always in the range of <10^−10^ mbar, operating with a monochromatic Al K_α_ X-ray source. Surveys and high resolution spectra were measured at pass energy of 100 eV and 20 eV, correspondingly. XPS analysis was performed with Unifit 2015 software^54^. Note that charging occurring from the photoelectron emission in XPS measurements have been compensated by an electron flood gun. Generally, charging effects lead to a slight broadening of the full width half maximum (FWHM) of the XPS peaks, while at the same time the electron beam emitted by the flood gun can modify surface organic species^55^,^56^, therefore care has to be taken in the interpretation of the data. Also by electron irradiation, XPS peaks may shift to lower or higher binding energies (BE)^55^, as well as changes in the oxidation state may occur (*cf*. Supplementary Fig. S3)^42^,^57^. Therefore here only for strongly charging native RE oxide films (without Tc), we have used an electron flood gun with the typical operating parameters: 2.2 mA and 2 V.

It is further important to note that numerous reports charge-correct the measured BE using the dominant C1s peak, attributing it to adventitious carbon and placing it into a range of 284.5–285.5 eV. This approach leads to inconsistencies in the energy positions between reports as the final charge-corrected binding energy is not accounting for the nature of different organic species^39^,^58^, as well as for differences in the chemical composition of the atmospheric air^29^. Therefore in this work the position of Gd 4d peak was taken as a reference for the charge correction.

Wettability of the films surface was characterized by the WCA measurements (Krüss goniometer, DSA 100). The mean value was calculated from at least five measured WCA values for each sample (water drop volume of 3 μl).

Morphological characterization was made by AFM (Nanosurf Easy Scan equipment). Using an Al-coated Silicon tip (type Tap190Al-G, BudgetSensors: resonance frequency 190 kHz, force constant 48 N/m, tip radius <10 nm, half cone angle at the apex 10°). The WSXM software^59^ was used to process the AFM images.

## Additional Information

**How to cite this article**: Külah, E. *et al*. Surface chemistry of rare-earth oxide surfaces at ambient conditions: reactions with water and hydrocarbons. *Sci. Rep.* **7**, 43369; doi: 10.1038/srep43369 (2017).

**Publisher's note:** Springer Nature remains neutral with regard to jurisdictional claims in published maps and institutional affiliations.

## Supplementary Material

Supplementary Information

## Figures and Tables

**Table 1:** Roughness analysis of Gd_2_O_3_ film in its native state and after evaporation of Tc.

Sample	State	rms roughness (nm)	Lateral correlation length (nm)
Gd_2_O_3_	fresh	0.6	19.8 ± 0.6
	aged	0.4	12.6 ± 0.2
Gd_2_O_3_ + 0.5 nm Tc	fresh	0.7	29.8 ± 0.2
	aged	3.2	27.0 ± 0.7
Gd_2_O_3_ + 2 nm Tc	fresh	2.2	26.6 ± 1.2
	aged	2.0	26.8 ± 0.7

**Table 2:** XPS atomic concentrations of *in-situ* prepared films (without braking vacuum): Gd2O3 surface in its native state and after evaporation of tetracene at two coverages.

Film	Total: Gd, %	Total: O, %	Total: C, %
Gd_2_O_3_ native	32.5	67.5	0
Gd_2_O_3_ + 0.5 nm Tc	29.6	50.5	19.9
Gd_2_O_3_ + 2 nm Tc	30.2	41.7	28.1
