# Supplementary material for: Surface chemistry of rare-earth oxide surfaces at ambient conditions: reactions with water and hydrocarbons
Source: Sci Rep. 2017 Mar 22;7:43369. doi: 10.1038/srep43369 (PMC5361147; doi:10.1038/srep43369)
Supplement: Supplementary Information [file srep43369-s1.docx]

***Supplementary Information***

**Surface chemistry of rare-earth oxide surfaces at ambient conditions: reactions with water and hydrocarbons**

Elçin Külah^1,†^, Laurent Marot^1^, Roland Steiner^1^, Andriy Romanyuk^2^, Thomas A. Jung^3^, Aneliia Wäckerlin^1,4,*^, Ernst Meyer^1,*^

^1^ Department of Physics, University of Basel, Klingelbergstrasse 82, 4056 Basel, Switzerland

^2^  Glas Trösch AG, Industriestrasse 29, 4922 Bützberg, Switzerland

^3^ Laboratory for Micro- and Nanotechnology, Paul Scherrer Institute, 5232 Villigen, Switzerland

^4^ Laboratory for Thin Films and Photovoltaics, Empa — Swiss Federal Laboratories for Materials Science and Technology, Überlandstrasse 129, CH-8600 Dübendorf, Switzerland

Present Address:

^†^ Advanced Semiconductor Quantum Materials, ETH Zürich, Otto-Stern-Weg 1, HPF E 18, CH-8093 Zürich

* Corresponding authors: aneliia.waeckerlin@empa.ch, ernst.meyer@unibas.ch

Table of contents

[1) Dissolution of the rare-earth oxides (REOs) in water 2](#_Toc469350500)

[2) Reaction of RE oxy-fluorides and oxy-nitrides with water and air 3](#_Toc469350501)

[3) A) Aging of CeO_2_ and Reduction from Ce(4+) to Ce(3+) 5](#_Toc469350502)

[B) Stability of CeO_2_ chemical composition over time under UHV conditions 9](#_Toc469350503)

[4) Surface analysis of aged-in-air REOs by XPS at 90°, 60° take-off angles 10](#_Toc469350504)

[5) Saturated chemical state of Gd_2_O_3_ film after exposure to air: native film versus tetracene-modified film 12](#_Toc469350505)

[6) Analysis of surface morphology 15](#_Toc469350506)

# Dissolution of the rare-earth oxides (REOs) in water

In previous studies concerning hydrophobicity of the rare-earth oxides (REOs), the hydrophobic behavior is explained on the basis of their particular electronic structure^1,2^. However, these inorganic REO compounds exhibit significant difference in the electronegativity (Δχ ~ 2.3) and are ionic, meaning strong Coulomb interaction with polar water molecules. Fact of strong interaction of REO with water points on the hydrophilic nature of the surface (see Fig. 2a-d in main article) and is contradictory to the Azimi et al. statement of their intrinsically water repellent behaviour. The observation that the REOs exhibit high water contact angles (WCA) of > 90° may be reproduced only upon storing samples for a long time in air, which leads to adsorption of hydrocarbons from air atmosphere^3^ (see Fig. 2 and Fig. 3 of the main article). As is shown in the main text, the films transform from oxide to hydroxide^4^- carbonate^5^ mixture after supply of water and CO_2_ during exposure to air. Nevertheless, transformation changes nothing with respect to the water reactivity of the REOs since any carbonate being an ionic compound is hydrophilic and strongly reacts with water^6^.

In the following chapter, the dissolution of the REOs is discussed. Oxides of Ce, Gd, Ho, Er, Tb were dipped into 1 l of deionized water for defined periods of time, till the films were fully dissolved. After each time step, X-ray photoelectron spectroscopy (XPS) and thickness measurements using ellipsometry and profilometery were performed. The XPS survey spectra of the investigated Ho_2_O_3_, Gd_2_O_3_ and Er_2_O_3_ are given as examples (see Fig. S1a-c). After 24 hours staying in water, the REO compounds fully dissolved and only silicon dioxide, coming from underlying glass substrate, was detected.

**
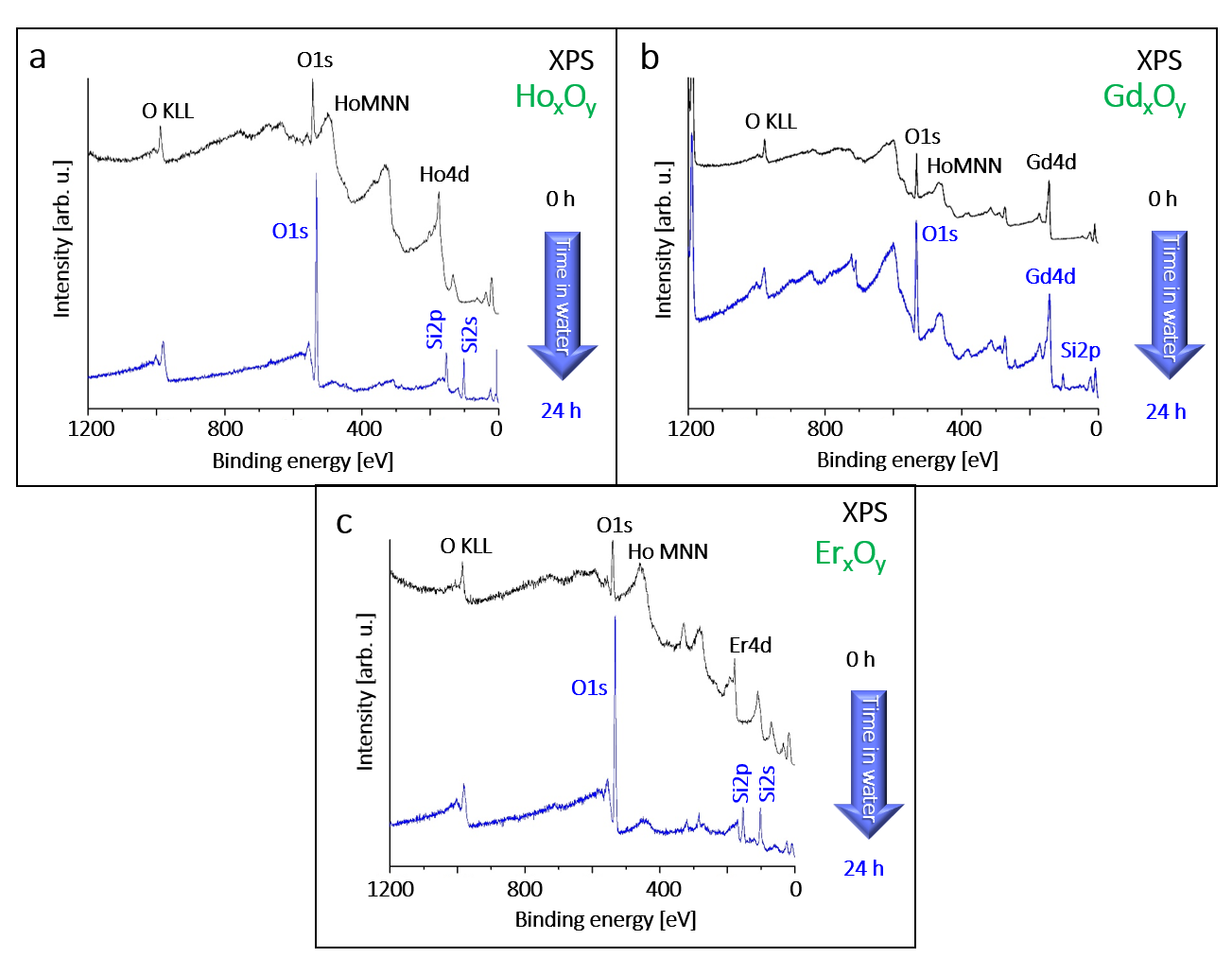
**

**Figure S1:** The rare-earth oxides, as highly reactive towards water materials. XPS normalized surveys for three different types of REO: Gd_2_O_3_, Ho_2_O_3_ and Er_2_O_3_ before (in-situ, fresh) and after (exposed to air, aged, 24 h dipped into 1 l deionized water) exposure to H_2_O. After 24 hours staying in deionized water, the peaks corresponding to RE element was decreased and peaks corresponding to Si increased. Appearance of Si peak, originating from the SiO_2_ substrate, clearly evidences a dissolution of the films in water.

# Reaction of RE oxy-fluorides and oxy-nitrides with water and air

In the context of water/RE surface interactions, we have also investigated other ionic RE compounds, where electronegativity difference between the ions of lanthanides (La) and the second ion in the compound is different, i.e. namely RE nitride and RE fluoride: Δχ (La-N) ~ 1.94 vs. Δχ (La-F) ~ 2.88. The films, prepared here, contain small amount of oxygen due to high reactivity of the compounds, thereby further referred as oxynitrides La_x_O_y_N_z_ and oxyfluorides La_x_O_y_F_z_. The exact composition of thin Gd_x_O_y_F_z_, Ce_x_O_y_F_z_ and Ce_x_O_y_N_z_ films (of 10, 21 and 24 nm thickness) was defined from XPS atomic concentration analysis and is listed in a Table S1(corresponding surveys are shown in Fig. 1 of main text and Fig. S2). The state of the film was controlled in i) the fresh state - directly after film preparation without breaking high vacuum (HV) conditions, ii) the aged state – after prolonged exposure to air, and iii) after prolonged exposure to water.

Film of Gd_x_O_y_F_z_ after 17 h in 1 l of deionized water revealed no Gd as detected by XPS, evidencing full dissolution. The Ce_x_O_y_F_z_ film after 1 h of exposure to water loses the fluorine, which content decreases from 32 at.% down to 13 at.% and simultaneously increase of the oxygen content from 23 at.% up to 38 at.%, evidencing a substitution of F by O. Longer exposure to water (24 h) leads to further F-substitution: 5 at.% F, 44 at.% O. After/During substitution of F, the Ce_x_O_y_ undergoes transformation to Ce-OH and finally, after ca. 235 h in water, film completely dissolves. The longer time, required for dissolution of Ce_x_O_y_F_z_ in comparison to dissolution of Gd_x_O_y_F_z_, can be related with the different kinetic/dynamic of the dissolution process, which is around two order of magnitude lower^7–9^.

Regarding to Ce oxy-nitride, the N content was diminished and substituted by O already after one day staying in air, even before the water dipping test was performed (see Fig. 1 in main article). According to the literature, RE nitrides supposed to show even higher water contact angles than the corresponding oxides, explained on the basis of lone electron pairs of N vs. O and corresponding H-bonding to water.^10^


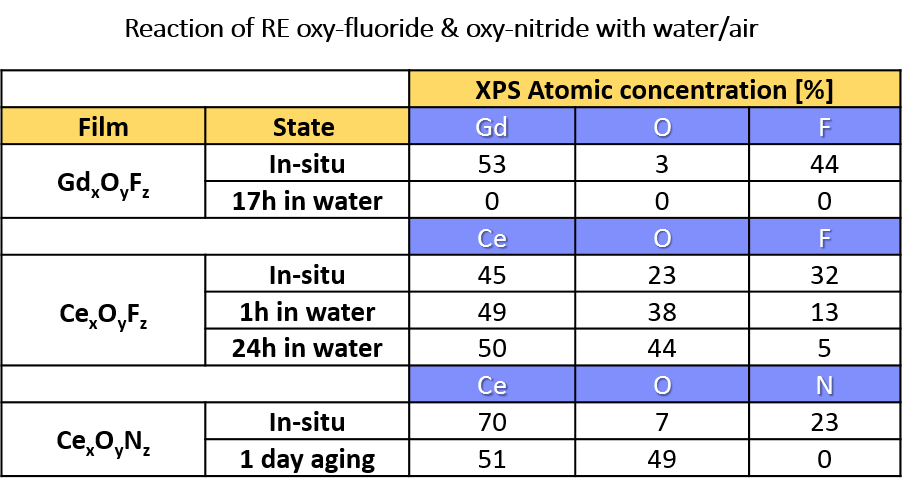


**Table S1:** Three different RE films, namely gadolinium-oxyfluoride, cerium-oxyfluoride and cerium-oxynitride, were investigated in their reaction with deionized water/moisture in air. The atomic concentrations of the films before and after contact with water/air are listed. Gd_x_O_y_F_z_ was fully dissolved after 17 h being dipped into 1 l of deionized water. Ce_x_O_y_F_z_ undergoes a substitution of F by O with the time in deionized water. Ce_x_O_y_N_z_ undergoes substitution of N by O already staying in air atmosphere. Finally, all films fully dissolve after defined time periods staying in deionized water.

Lastly, additional XPS surveys are shown for Gd_x_O_y_F_z_ film in-situ directly after preparation (Fig. S2 - black curve) and after 17 h in the deionized water (see Fig. S2 - blue curve). After 17 h in water only SiO_2_ was detected, coming from the glass substrate itself.


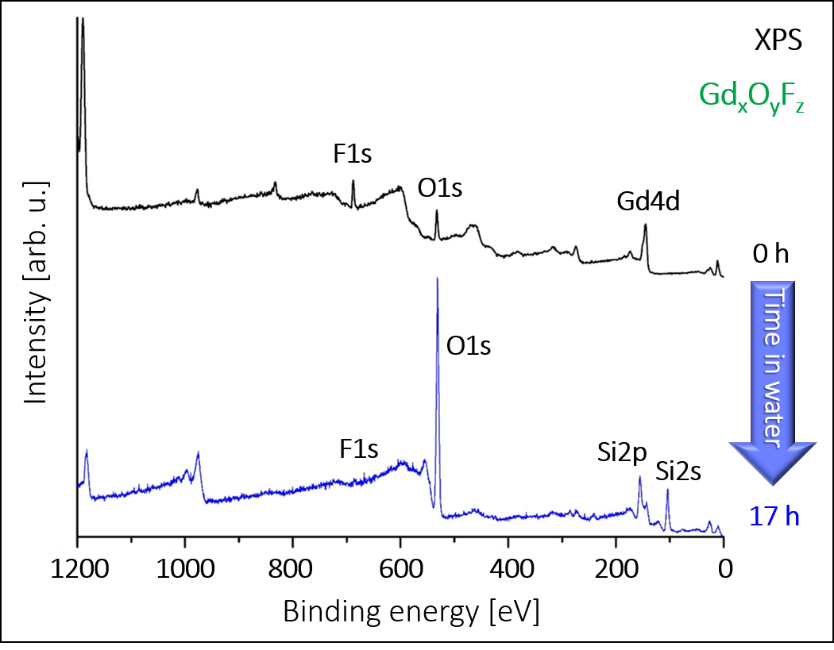


**Figure S2:** XPS survey spectra for a gadolinium oxy-fluoride thin film (10 nm) on SiO_2_-coated float glass substrate. In-situ (black curve) and after 17 h in deionized water (blue curve) are plotted. It is clearly seen that no more F and Gd are observed after film has been dipped in water, the Si2p respectively Si2s peaks appear from the under-laying glass substrate.

# A) Aging of CeO_2_ and Reduction from Ce(4+) to Ce(3+)

In-situ XPS measurements were performed for the cerium oxide films directly after film deposition in HV system without breaking the vacuum. Afterwards, the films were exposed to air and were undergoing aging during prolonged time (24 h). Subsequently XPS measurements were repeated before and after Ar sputtering for extended analysis of the existing catalytic reactions at the films surface when being exposed to air.

Ce3d, O1s and C1s core level spectra are depicted in Fig. S3 a)-c) for a 10 nm cerium oxide film. Following classification was done: i) in-situ characterization of the film, represented by black curves, ii) after air exposure for 24 h aging, marked in blue, and iii) after Ar sputtering of the 24 h aged-in-air film at UHV conditions, marked in green. All spectra were fitted with the minimal amount of components necessary to obtain a good fit. The corresponding Ce3d XPS peaks belonging to both oxidation states (3+, 4+) differ clearly in their shape and components, described elsewhere^11,12^. Ce3d peak was taken as reference peak since the REO film exhibit high charging effect while measuring XPS. The reason for Ce as reference - specific components for 3+ and 4+ can be clearly distinguished between each other and thereby the spectra can be aligned. This energy alignment results also into reasonable BE for C1s and O1s. Besides, there is always a discussion in literature about proper energy referencing, which must be carefully analyzed for each case.

In case of our herein shown example of a 10 nm Ce oxide film, the Ce3d peak was fitted by defined components and parameters given in literature as: the multiple components (6) and their shape (black curve in Fig. S3 a)), containing v, v^II^, v^III^, u, u^II^ and u^III^ components corresponds to the 4+ oxidation state of the film^12^. The smaller contribution, marked by v_o,_ v^I^ and u^I^, correspond to Ce in the 3+ oxidation state^12^. The two contributions from the 3+ and 4+ results also in two components for O1s peak, where the component at 528.7 eV belongs to Ce(III) and the second (highest) component at 530.9 eV represents the Ce(IV) state (see Fig. S3 b) - black). Small amount of Ce-OH was detected at ~ 532.5 eV^13^, no carbon was present (Fig. S3c)).

After 24 h exposure of film to air, the characteristic features and shape of the Ce3d line changes, i.e. the components corresponding to 3+ (v^I^ and u^I^) significantly increase pointing on the partial reduction of Ce(IV) to Ce(III) evidencing catalytic reaction happening at the film’s surface (Fig. S3 a), blue curve for 24 hours in air atmosphere)^12^. H_2_O splitting^14^, as well as carbonates / carboxylates formation due to CO/CO_2_ adsorption^5^ occur at the films surface. Moreover, the film transforms partially from its pure oxide state to a carbonated hydroxide when reacting in air atmosphere, which is already given in previous studies by others for the light rare earth oxides^15^. As an additional comment: the hydration process is compared to the carbonation process limited to the outer layers of the REOs^15^. Therefore, our fit of the Ce3d peak given for 24 h aging (Fig. S3a), blue) with its in total 8 components – used normally for the (pure) oxide state of Ce – is not fully representing the real film state since it occurs in a mixed form combined of: i) oxide in 3+ as well as 4+, ii) hydroxide, and iii) carbonate/carboxylate. However, it was quite difficult to perform such a fitting including all appearing species of the aged-in-air cerium oxide film, thereby a simplified fit was performed. Nevertheless, the present fit depicts at least quite well the transformation of the oxidation state from Ce(IV) to Ce(III)^12^. The reduction can result from adsorbed CO, oxidation of airborne hydrocarbons and resulting carbonate formation.^5^ The presence of water tends, however, to oxidize of cerium oxide back to Ce(4+), CeO_2_^16^ with corresponding formation of hydroxide phase^14^. Thereby, at the air-exposed film two phases co-exist.

Further, corresponding O1s peak after 24 h aging in air lead to multiple components, i.e. at 528 eV staming from Ce(III)-O species (Fig. S3b) - blue), as a result of the partial reduction of the predominant CeO_2_ in-situ film, at 529.5 eV appears from Ce(IV)-O. Furthermore, the hydroxide formation is visible at a binding energy of 530.9 eV, followed by the C-O species at 531.9 eV. The Ce carbonate/carboxylate is placed at 532.9 eV, + 1 eV with respect to C-O component. Little contribution of water at the highest binding energy of 534 eV was detected as well. The overall different components of O1s, appeared after sample being in air, were fitted with minimum amount of peaks corresponding to oxide, hydroxide and carbonate/carboxylate of Ce, adsorbed water. The chemical bonds stemming from these phases have different relative binding energy and are placed with the following shifts towards higher binding energy (with respect to oxide): a hydroxide + 1 - 2 eV, carboxylate COOH ~ + 2 - 3 eV, carbonate CO_3_ ~ + 3 - 4 eV, adsorbed H_2_0 ~ + 4 -5 eV^17,4,18^. The corresponding C1s components: i) Ce-C for lowest binding energy of 283 eV, ii) C-C of 284.8 eV (+ 1.8 eV), and iii) Ce-CO_3_ of 288.7 eV (+ 5.7 eV) (Fig. S3c), blue). To summarize the exposure of CeO_2_ for 24 h to air partially reduces Ce(IV) to Ce(III), where the mixed phase of hydroxide and carbonate/carboxylate is formed.

Short Ar sputtering (performed only till C1s peak was diminished) lead to significant change in the Ce3d peak shape, evidencing full reduction from the mixed 3+ and 4+ phases to only one phase of 3+ oxidation state (see Fig. S3 c) - green)^12^. The complete reduction of the core level from Ce(III)/Ce(IV) to Ce(III) can be also seen explicitly in the corresponding O1s peak, where only remaining Ce(III)-O bonding at 528.2 eV is visible (Fig. S3b), green). Hydroxide (represented at 529.9 eV) and Ce carbonate/carboxylate (at 531.5 eV) were detected as well, whereby more hydroxide formation than carbonation process can be deduced from of XPS atomic concentrations. The higher hydroxide amount, detected after sputtering, comes from the fact of carbonation is limited only to the outer layers of the oxides, compared to the hydration of the REO ^15^. Reduction of cerium from Ce(IV) to Ce(III) after sputtering was observed as well by others, coming from the impact of Ar sputtering^19,20^. Besides, not only Ar ions, but also X-ray irradiation after prolonged time can reduce REO core level^21^. Thereby one has to analyze the chemical state of Ce in the film only in its ‘natural’ form and without ‘damaging’/reducing the sample by Ar sputtering.


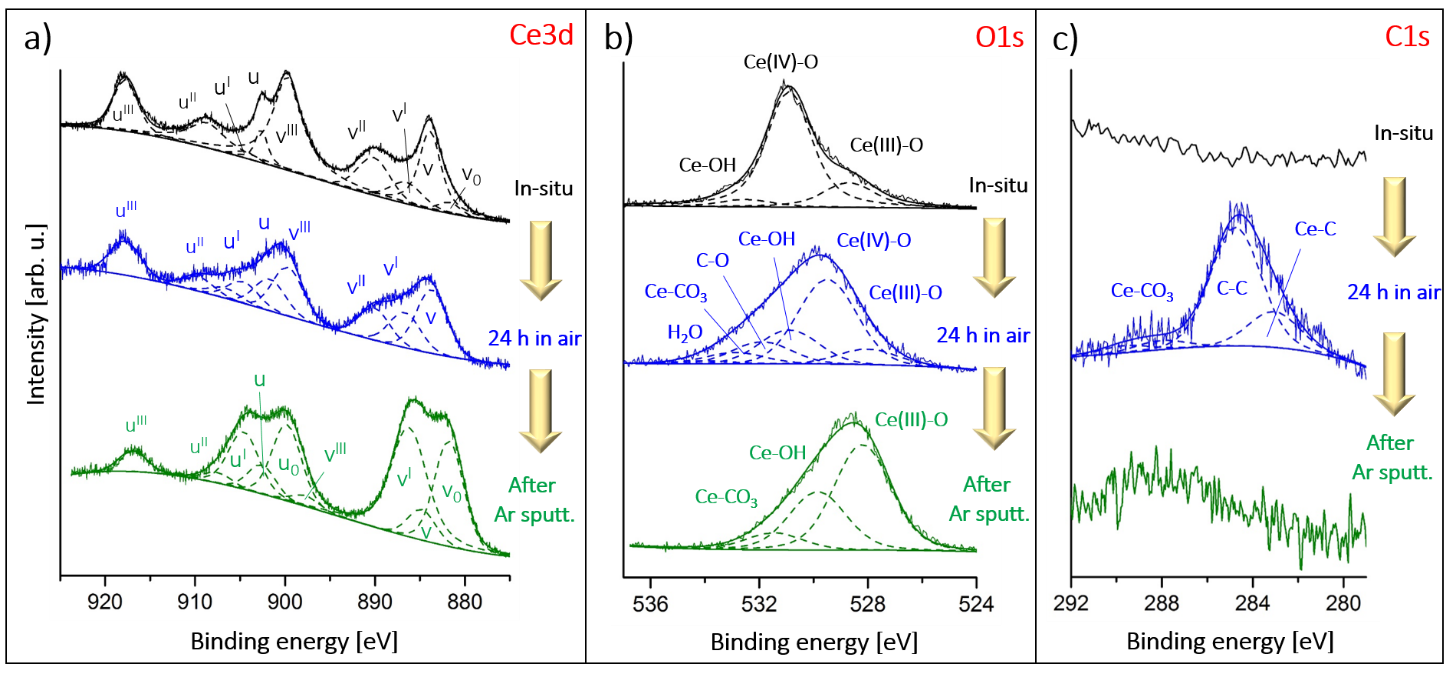


**Figure S3:** Normalized high resolution XPS spectra for a 10 nm thin cerium oxide film in its fresh (in-situ, black), aged (24 h in air atmosphere, blue) state and after Ar sputtering of the aged-in-air film (green). After 24 h aging, the film transforms from oxide phase (CeO_2_) to a mixture of 3+/4+ oxide, hydroxide and carbonate/carboxylate (blue curves from a)-c)). However, a significant modification of the Ce3d core levels occurs after Ar sputtering: a full reduction of Ce from 3+/4+ to only 3+ was deduced by the impact of Ar ions in UHV conditions, which can be clearly seen in the shape change of the Ce3d peak (green curve in a)). Besides, no more Ce(IV)-O bonding was detected for the corresponding oxygen peak given in part b), green spectra.

# B) Stability of CeO_2_ chemical composition over time under UHV conditions

A cerium oxide film of ca. 10 nm thickness was prepared at HV conditions (see section Methods – sample preparation in main article for detailed film preparation) and subsequent XPS measurements were done directly after deposition without breaking the vacuum. Afterwards, the film stayed for 14 hours in the XPS chamber at p ~ 10^-10^ mbar and then was measured again.

The Ce3d and O1s peaks after 0 hours in-situ (directly after deposition) exhibit a mixture of Ce(IV) and Ce(III) oxide, where Ce(IV) dominates. The shape and exact fit parameters for fit of the Ce3d peak are described elsewhere^12^. The O1s components referring to Ce(III) is located at 529.2 eV and to Ce(IV) at 530.9 eV (Fig. S4 a, b - black). Besides, small contribution of Ce-OH was detected in the oxygen peak at about 532.6 eV coming from reaction with water, present in small amount in the sputter chamber during the deposition process.

Keeping the sample over 14 hours at p ~ 10^-10^ mbar does not induce any change in Ce3d or O1s peaks (Fig. S4 a, b - blue). The peak components, calculated atomic percentages, but also the peak shapes are identical for both samples. It is important to note that intrinsic hydrophobic properties of REO have been reported by Khan et al.^2^, where the film needed to undergo 14h of ‘relaxation’ of the surface in vacuum in order to reach a hydrophobic state. The main argument was a modification of the O/Ce ratio of a Ce oxide film, which was probably either not carefully fitted or the surface has been really modified by adsorbates/contaminants coming from the chamber. In our case CeO_2_ film was always hydrophilic (measured directly after taking out from UHV system) and hydrophilicity was independent from amount of hours being the sample in vacuum. No change with respect to the film`s chemical state and thus correlated hydrophobicity was observed. The hydrophobic state of the film was always reached only after prolonged stay in air, as was analogously observed for all other prepared REOs surfaces (see Fig. 2 in main article).


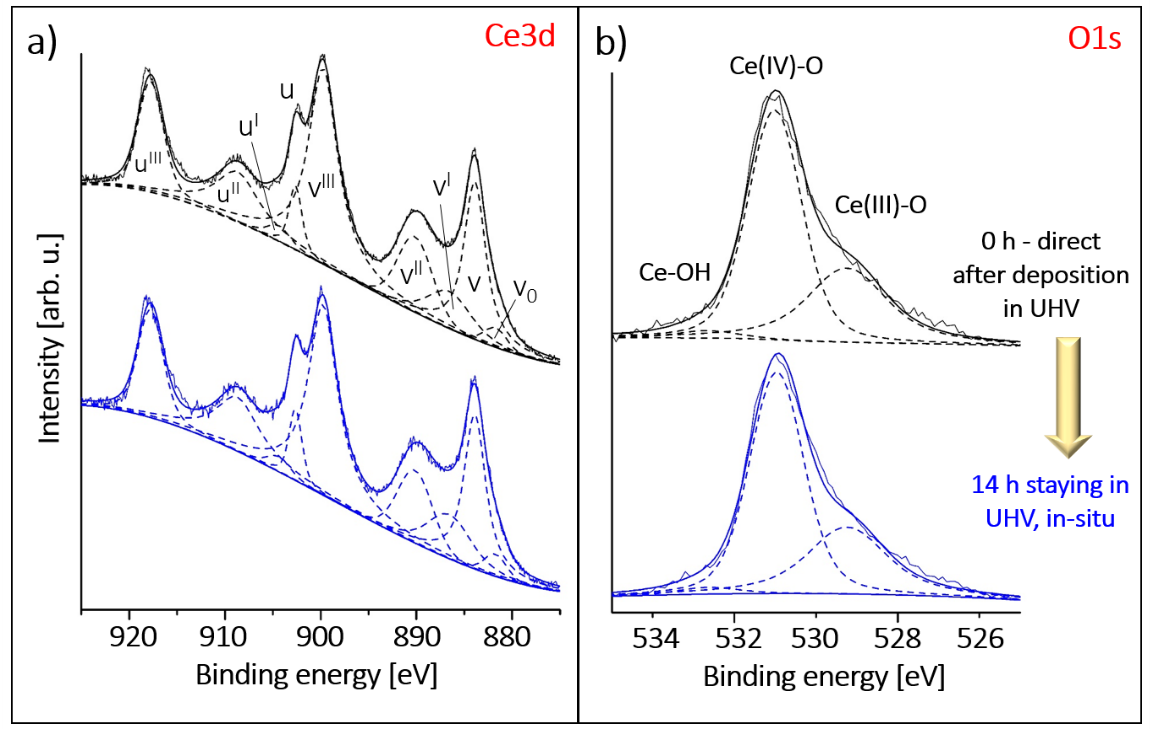


**Figure S4:** High resolution XPS spectra for a mixed 3+/4+ cerium oxide film (10 nm) shown in two in-situ states: i) 0 hours, meaning directly after film deposition in HV system (black), and ii) staying 14 hours in UHV and afterwards being measured (blue). Both states reveal identical spectra for the Ce3d (a) as well as for the O1s (b), evidencing no modification of the chemical state.

# Surface analysis of aged-in-air REOs by XPS at 90°, 60° take-off angles

Following reaction steps were evidenced for the REO films after being exposed to air atmosphere from HV system: i) transformation from oxide to hydroxide^4^ (Fig. S3 & Fig. 4 in main article), ii) formation of carbonates or carboxylates^5^ (Fig. 4 in main article), and iii) oxidation of hydrocarbons^5^. In order to investigate in details an aged-in-air (~ two month) Gd_2_O_3_ film surface, XPS at different take-off angles was measured. In case of a take-off angle equal 90°, i.e. perpendicular to the film surface, types of bonds and corresponding concentrations were analyzed for the O1s and C1s peaks (Fig. S4 b), c) - black curves). All spectra are fitted with minimal amount of components necessary to obtain a good fit and the charge correction was made by alignment of Gd 4d peak at 144.8 eV. Thereby the components and their binding energies must be considered relative to each other.

The Gd4d peak was fitted by six singlet peaks.^22^ The first five peaks belong to the so-called 9D component, whereas the broad last single peak at around 150.7 eV reflects its 7D component. Important to comment: the fitting parameters of the Gd4d are defined for a pure oxide with bigger full-width half maximum (FWHM), compared to the in-situ native oxide film, and therefore may not completely describe the present case, since the film is a mixture of oxide/hydroxide and carbonate after aging-in-air. The fit has to consist actually of six singlets for each of the Gd phases, contained in the mixed RE film (Gd-O, Gd-OH, Gd-CO_3_).

The O1s component at a binding energy of 534.3 eV (+ 4.3 eV) represents the most dominant component within the oxygen peak and corresponds to water, followed Gd-CO_3_ at 533.6 eV (+ 3.6 eV), by C-O at 532.4 eV (+ 2.4 eV), Gd-OH at 531 eV (+ 1 eV), and lastly with Gd-O at 530 eV (0 eV) as the smallest contribution. Among the carbon components, one can distinguish C-O bond at 287.5 eV with the most significant contribution, representative of around half of the whole C1s peak being 55.4 at.% from XPS concentration (Fig. S4c). Quantitatively, CO_3_ (292.5 eV, 21.5 at.%) and C-C (285.7 eV, 12.8 at.%) are second major components, and C-COOH (290.7 eV) provides the smallest concentration of 10.3 at.% out of 100 % of C1s.

When measuring with the take-off angle to 60°, one increases the surface sensitivity by collecting photoelectrons from shallower angle, increasing the signal coming from the top layers of the surface. The component concentrations in O1s as well as in C1s peak (slightly) differ, namely: i) concentration, calculated from O1s, of Gd-CO_3_ is increased from 20.2 at.%, 90° to 30.6 at.%, 60°, calculated from the films atomic percentage, ii) with respect to C1s peak, concentrations of CO_3_ and C-C increased from 21.5 at.% (90°) to 25.1 at.% (60°) and 12.8 at.% (90°) to 15.6 at.% (60°) respectively, whereby C-O remained the same and C-OOH clearly decreased from 10.3 at.% (90°) to 3.1 at.% (60°), iii) lower peak-to-peak ratio between C-O and CO_3_ calculated from the C1s spectra. Thus leading to the conclusion water and carbonate being placed at the top-most surface layers, and Gd hydroxide and Gd oxide being localized deeper inside, correspondingly. The trend of carbonate phase being localized at the very-top layers was already reported earlier^15^. The only ‘slight’ change in the concentration ratio stems from the rough surface (~ 1.5 nm, according to AFM data).


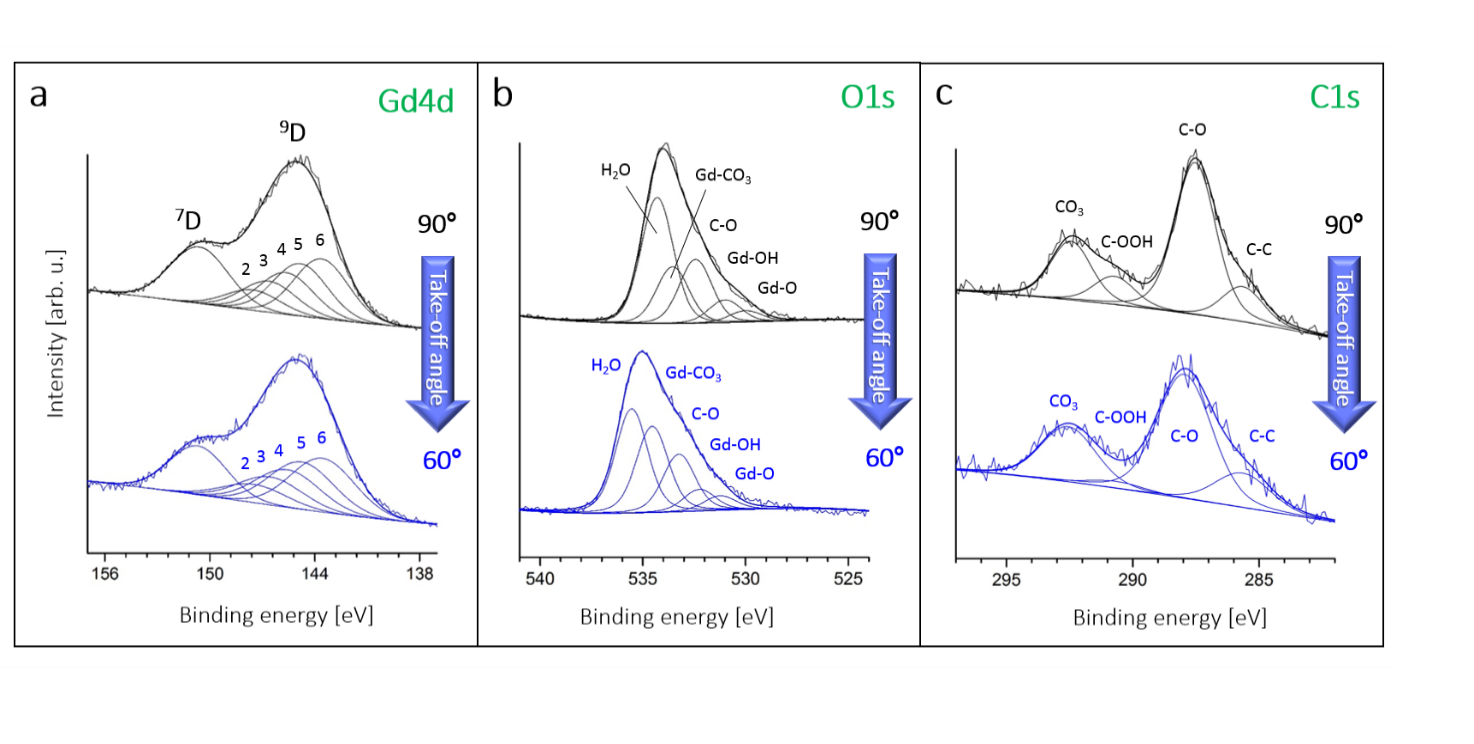


**Figure S5:** XPS Gd4d, O1s and C1s at different take-off angles (90°, 60°) for a 10 nm Gd oxide film, aged-in-air for ~ 2 month. It was observed that while going to shallower angle, relative contributions from H_2_O, Gd-CO_3_ increased and of Gd-OH, C-O and Gd-O decreased evidencing that a carbonation process occurring at Gd_2_O_3_ surface in air take place at the outer layers of the surface, then follows hydroxide phase propagating more into the bulk, and only then an oxide layer.

# Saturated chemical state of Gd_2_O_3_ film after exposure to air: native film versus tetracene-modified film

The several reaction processes at the REO surfaces were already discussed in the main text as well as in earlier paragraphs of SI, namely – hydroxylation, formation of carbonates / carboxylates, oxidation of hydrocarbons, - when being exposed to air and compared with native oxide surface (directly after preparation in-situ).

Further analysis of hydrocarbon interaction with RE oxide surface at RT was conducted, where tetracene molecules (C_18_H_12_) were evaporated on a Gd_2_O_3_ surface. Afterwards samples were exposed to air and left for 28 days (native Gd_2_O_3_) and 64 days (tetracene-covered Gd_2_O_3_) the surface analysis was performed via XPS.

Here, extended analysis of different C1s components before and after exposure to air at Gd_2_O_3_ surface is given below (see Table S2). To start with the native Gd oxide, the film revealed already in its in-situ state presence of Gd hydroxide (12.8 at.%). This is a result of small amount of water coming from the sputter chamber during film deposition forming additional hydroxide species beside pure Gd oxide. When aging in air for 64 days, carbonate/carboxylate formation beside hydroxide/oxide occur and water at the surface was detected (Table S2).

The lower concentration of Gd-OH (5 at.%) than Gd-CO_3_ (12.4 at.%) remains from the fact that the carbonation process is compared to the hydration of the REO films limited to the outer layers of the oxides, consistent with the limited penetration depth of the photoelectron through the layer, where the OH bonds are deeper located with respect to the topmost second layer of carbonate^15^.

The change in the chemical composition of the Gd_2_O_3_ surface is observed after deposition of C_18_H_12_ at two coverages: 0.5 nm Tc and 2 nm Tc. Amount of C-C bonds, which was detected in XPS directly after evaporation, reaches 12.5 % for 0.5 nm Tc and 25.6 at.% for 2nm Tc(Table S2). Remarkable change in the corresponding (in-situ) O1s spectra of the C_18_H_12_-covered films compared to the native (in-situ) Gd oxide was observed: increase in binding energy (+ 1.3 eV with respect to the highest component of the peaks) (see discussion in the main text of the article). The consumption of O from the surface after evaporation of tetracene evidences a reduction of the Gd_2_O_3_ surface. Presence of C-O bonds, observed in C1s evidence a partial oxidation of tetracene. See also a discussion in the main text of the manuscript.

After aging for 28 days of both tetracene-covered films exhibited very similar elemental composition, independently from the starting Tc coverage(Table S2). C-C bonds for both aged films amount to ~6 at.%. We assume that the final ‘saturated’ film is not more catalytically active and requires heat in order to decompose carbonate layer, recover oxide state and reactivate the surface, as it is done in the catalytic convertors of vehicles.


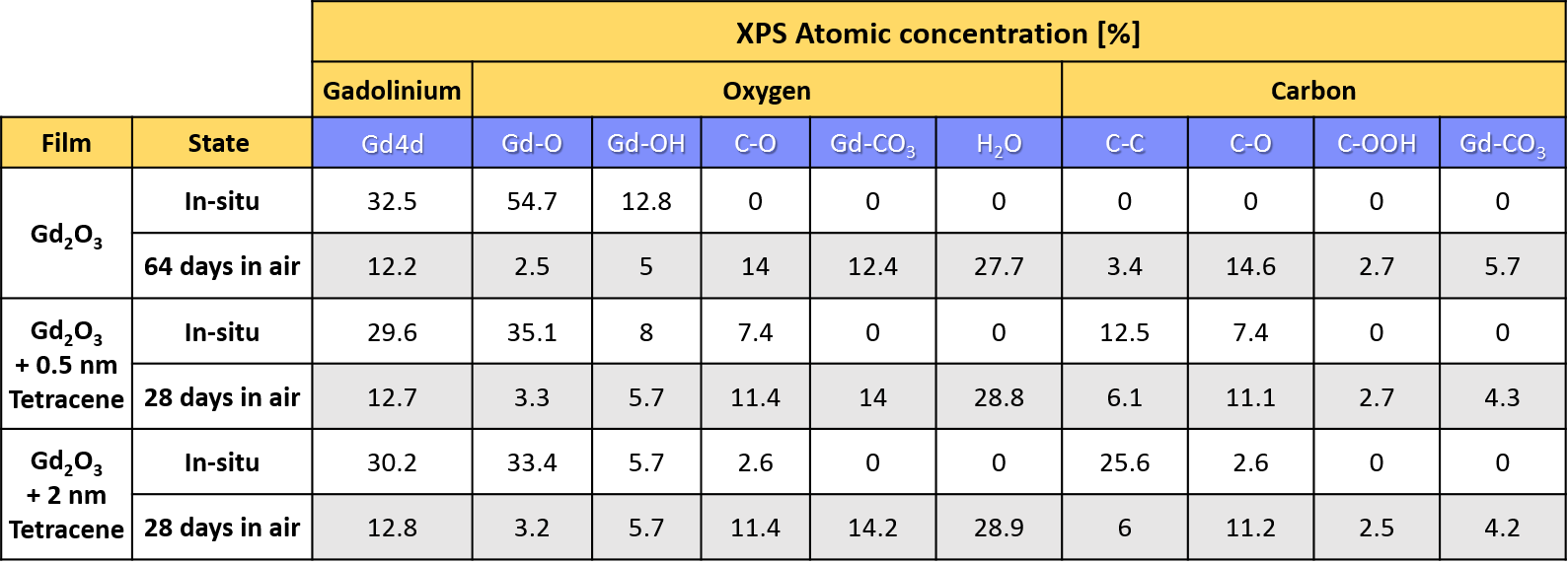


**Table S2:** The atomic concentrations of one native and two tetracene(C_18_H_12_)-covered Gd_2_O_3_ films in their in-situ (fresh) and aged-in-air state are listed. Amount of C-C species is significantly reduced for the tetracene-covered films after aging for 28 days. The diminution of C-C and increase in C-O amount reveals the decomposition and/or conversion of the molecules due to catalytic reactions of the REO surface in air atmosphere. After aging, all films – native and C_18_H_12_-covered Gd oxides – exhibit the same chemical composition independently from the starting condition of the hydrocarbon at the film surface, pointing on the favored ‘saturated’ state of the film.

# Analysis of surface morphology

The morphology of the Gd_2_O_3_ films depends on the starting conditions: native film vs. Tc covered film. The vertical and lateral roughness was determined from root mean square (RMS) values and lateral correlation function. In order to determine a lateral correlation length, the Height-Height Correlation Function (HHCF) was obtained from the AFM images (Fig. 3 of main text, Fig. S6) with Gwyddion Software and fitted to a model

$g'\left( r \right)=2\sigma^{2}\left( 1- e^{{-\left( \frac{r}{\xi} \right)}^{2\alpha}} \right)$,

where $\sigma$ corresponds to the surface roughness, $\xi$ is the correlation length and α is the Hust parameter.^23^ The *r* parameter was set from 0 to 260 nm for all of our fits to calculate the lateral correlation length, since HHCF saturates already < 260 nm (Fig.S6, black dots - measured data points, red line - fit of HHCF). The obtained values of lateral correlation length are summarized in the Table 1 of main text.


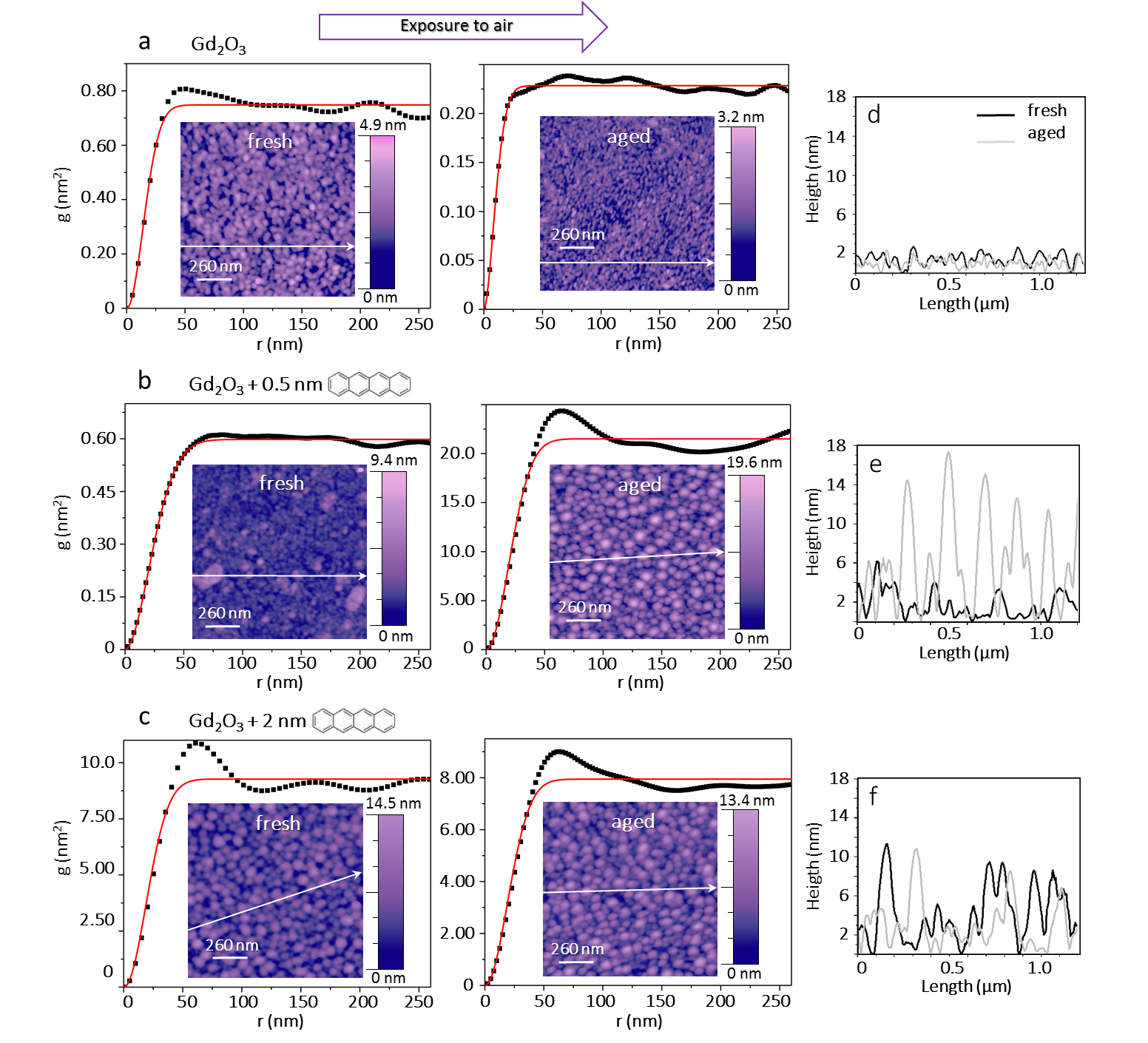


**Figure S6:** AFM images of Gd_2_O_3_ surface in fresh (left) and aged (right) state with corresponding Height-Height Correlation Functions and fit of lateral correlation length: (a) native Gd_2_O_3_, (b) Gd_2_O_3_ + 0.5 nm Tc, (c) Gd_2_O_3_ + 2 nm Tc.

**References:**

1. Azimi, G., Dhiman, R., Kwon, H.-M., Paxson, A. T. & Varanasi, K. K. Hydrophobicity of rare-earth oxide ceramics. *Nat. Mater.* **12,** 315–320 (2013).

2. Khan, S., Azimi, G., Yildiz, B. & Varanasi, K. K. Role of surface oxygen-to-metal ratio on the wettability of rare-earth oxides. *Appl. Phys. Lett.* **106,** 061601 (2015).

3. Preston, D. J. *et al.* Effect of hydrocarbon adsorption on the wettability of rare earth oxide ceramics. *Appl. Phys. Lett.* **105,** 011601 (2014).

4. Cohen, S. *et al.* The interaction of H2O with the surface of polycrystalline gadolinium at the temperature range of 300–570K. *Surf. Sci.* **617,** 29–35 (2013).

5. Trovarelli, A. Catalytic Properties of Ceria and CeO _2_ -Containing Materials. *Catal. Rev.* **38,** 439–520 (1996).

6. Jeong, S.-B., Yang, Y.-C., Chae, Y.-B. & Kim, B.-G. Characteristics of the Treated Ground Calcium Carbonate Powder with Stearic Acid Using the Dry Process Coating System. *Mater. Trans.* **50,** 409–414 (2009).

7. Moeller, T. & Kremers, H. E. The Basicity Characteristics of Scandium, Yttrium, and the Rare Earth Elements. *Chem. Rev.* **37,** 97–159 (1945).

8. Mioduski, T., Gumiński, C. & Zeng, D. IUPAC-NIST Solubility Data Series. 100. Rare Earth Metal Fluorides in Water and Aqueous Systems. Part 1. Scandium Group (Sc, Y, La). *J. Phys. Chem. Ref. Data* **43,** 013105 (2014).

9. Mioduski, T., Gumiński, C. & Zeng, D. IUPAC-NIST Solubility Data Series. 100. Rare Earth Metal Fluorides in Water and Aqueous Systems. Part 2. Light Lanthanides (Ce–Eu). *J. Phys. Chem. Ref. Data* **44,** 013102 (2015).

10. Zenkin, S., Kos, Š. & Musil, J. Hydrophobicity of Thin Films of Compounds of Low-Electronegativity Metals. *J. Am. Ceram. Soc.* **97,** 2713–2717 (2014).

11. Zhang, F., Wang, P., Koberstein, J., Khalid, S. & Chan, S.-W. Cerium oxidation state in ceria nanoparticles studied with X-ray photoelectron spectroscopy and absorption near edge spectroscopy. *Surf. Sci.* **563,** 74–82 (2004).

12. Preisler, E. J., Marsh, O. J., Beach, R. A. & McGill, T. C. Stability of cerium oxide on silicon studied by x-ray photoelectron spectroscopy. *J. Vac. Sci. Technol. B Microelectron. Nanometer Struct.* **19,** 1611 (2001).

13. Mullins, D. ., Overbury, S. . & Huntley, D. . Electron spectroscopy of single crystal and polycrystalline cerium oxide surfaces. *Surf. Sci.* **409,** 307–319 (1998).

14. Mullins, D. R. *et al.* Water Dissociation on CeO _2_ (100) and CeO _2_ (111) Thin Films. *J. Phys. Chem. C* **116,** 19419–19428 (2012).

15. Adachi, G. & Imanaka, N. The Binary Rare Earth Oxides. *Chem. Rev.* **98,** 1479–1514 (1998).

16. Padeste, C., Cant, N. W. & Trimm, D. L. The influence of water on the reduction and reoxidation of ceria. *Catal. Lett.* **18,** 305–316 (1993).

17. Abi-aad, E., Bechara, R., Grimblot, J. & Aboukais, A. Preparation and characterization of ceria under an oxidizing atmosphere. Thermal analysis, XPS, and EPR study. *Chem. Mater.* **5,** 793–797 (1993).

18. Zenkevich, A., Lebedinskii, Y., Scarel, G. & Fanciulli, M. in *Defects in High-k Gate Dielectric Stacks* (ed. Gusev, E.) **220,** 147–160 (Kluwer Academic Publishers, 2006).

19. Holgado, J. ., Munuera, G., Espinós, J. . & González-Elipe, A. . XPS study of oxidation processes of CeOx defective layers. *Appl. Surf. Sci.* **158,** 164–171 (2000).

20. Qiu, L., Liu, F., Zhao, L., Ma, Y. & Yao, J. Comparative XPS study of surface reduction for nanocrystalline and microcrystalline ceria powder. *Appl. Surf. Sci.* **252,** 4931–4935 (2006).

21. Paparazzo, E. XPS studies of damage induced by X-ray irradiation on CeO2 surfaces. *Surf. Sci.* **234,** L253–L258 (1990).

22. Thiede, T. B. *et al.* Evaluation of Homoleptic Guanidinate and Amidinate Complexes of Gadolinium and Dysprosium for MOCVD of Rare-Earth Nitride Thin Films. *Chem. Mater.* **23,** 1430–1440 (2011).

23. Gredig, T., Silverstein, E. A. & Byrne, M. P. Height-Height Correlation Function to Determine Grain Size in Iron Phthalocyanine Thin Films. *J. Phys. Conf. Ser.* **417,** 012069 (2013).
